# Background fish feminization effects in European remote sites

**DOI:** 10.1038/srep11292

**Published:** 2015-06-10

**Authors:** Sergio Jarque, Laia Quirós, Joan O. Grimalt, Eva Gallego, Jordi Catalan, Reinhard Lackner, Benjamin Piña

**Affiliations:** 1 Institute of Environmental Assessment and Water Research (IDÆA-CSIC). Jordi Girona, 18. 08034 Barcelona, Catalonia, Spain; 2 CREAF. Edifici C, Campus de Bellaterra. Autonomous University of Barcelona. 08193 Cerdanyola del Vallès, Catalonia, Spain; 3 Institute of Zoology, University of Innsbruck, Technikerstrasse 25, Innsbruck, A-6020, Austria

## Abstract

Human activity has spread trace amounts of chemically stable endocrine-disrupting pollutants throughout the biosphere. These compounds have generated a background level of estrogenic activity that needs to be assessed. Fish are adequate sentinels for feminization effects as male specimens are more sensitive than humans to exogenous estrogenic compounds. High mountain lakes, the most distant environments of continental areas, only receive semi-volatile compounds from atmospheric deposition. We analyzed the expression levels of estrogen-regulated genes in male fish from these mountain lakes in Europe. Incipient feminization involving expression of estrogen receptor and zona radiata genes revealed a widespread diffuse estrogenic impact. This effect was correlated with the concentrations of some organochlorine compounds in fish and was consistent with the persistent occurrence of these tropospheric pollutants in the most remote planet regions. These results should be of general concern given the increasing endocrine disruption effects in human populations.

The accumulated toxicity of the exogenous endocrine disrupting compounds (EDCs) distributed throughout the planet has been overlooked so far^1^. EDCs are chemical pollutants that can penetrate into exposed biota and alter their endocrine system by mimicking or counteracting the action of natural hormones^2^. These alterations may lead to multiple deleterious effects, from sterility to mental deficiencies, and to a variety of developmental defects^2^,^3^,^4^,^5^, which may be triggered by extremely low environmental concentrations, some times in the sub-nM range. Many EDCs are pervasive and bioaccumulate along trophic chains, which increases their potential toxicity^5^.

Fish constitute particularly adequate sentinels for feminization effects, as male specimens respond readily to the presence of estrogens in the blood stream and show estrogen-responsive gene expression in liver^6^. Among all fish species, *Salmonidae* are especially suited to detect initial feminization effects, as at least three genes are known to respond strongly to estrogen in liver: vitellogenin (Vtg) and two zona radiata protein (Zrp) genes. In the present study we are adding the estrogen receptor (ER) gene to this list. Those EDCs that mimic the female steroid hormone estradiol bind to the ER, the key factor that elicits estrogenic response in vertebrates.

Detection of the female-specific egg-forming Vtg and Zrp proteins in blood and liver samples from male fish requires specific antibodies for each fish species and relatively large sample amounts obtained either by exsanguination (blood) or dissection (liver)^7^,^8^,^9^,^10^,^11^. An alternative to immunodetection of these proteins is the analysis of the corresponding messenger RNA (mRNA) in liver^12^,^13^,^14^. Synthesis of mRNA of estrogen-regulated genes is the primary molecular response to estrogenic compound intake and precedes translation and secretion into the bloodstream of the corresponding proteins^6^. Its detection and quantitation only requires very small amounts of sample and benefits from the versatility and sensitivity of polymerase chain reaction (PCR)- based procedures^15^ which allows analysis of genes whose protein products are present at very low concentrations in the cell, as it is the case of the ER. For comparative purposes, the mRNA expression for cytochrome P450 1A (Cyp 1A) that records the ectopic activation of the receptor of dioxin-like compounds^16^,^17^ has also been measured.

Total RNA isolated from different liver *Salmo trutta* (39 males and 61 females) collected in lakes from two mutually distant European mountain ranges, the Tatras and the Pyrenees^16^ (Supplementary Table S1) were retrotranscribed and used as template in a standard PCR using appropriate oligonucleotide pairs to detect Vtg, Zrp and ERα (Estrogen Receptor α) mRNA in liver. The reaction produced DNA fragments (amplicons) of the expected size for the three genes (not shown) and whose sequences were identical or very similar to the genes used for the primer design: 100% identity to the published *S. trutta* VtgA for the Vtg amplicon, 97% identity to *Salvelinus alpinus* zona pellucida protein beta for the Zrp1 amplicon, and 100% identity to *Salmo salar* ERα for the ERa amplicon. In a preliminary validation experiment, mRNA levels of these three genes were compared to the reference gene ß-Actin mRNA levels^17^ in control and estradiol-treated juvenile brown trout by qRT-PCR, using the same set of primers. As shown in Fig. 1A, the treatment increased hepatic mRNA levels for the three genes, corresponding the strongest induction to the Vtg gene (more than 100 fold) and the lowest one to ERa (less than 10-fold). These results indicate that the three genes can be used to monitor the exposure of *S. trutta* to estrogens.

Hepatic mRNA levels of estrogenic-responsive genes in natural populations of *S. trutta* showed variations of five to seven orders of magnitude, following distinct patterns in male and female specimens. Females with undifferentiated ovaries or only incipient oogenesis (reproductive stages 1 and 2) showed very low Vtg and Zrp1 mRNA levels (Fig. 1B) comparable to typical levels in males (Table S1). Expression of both genes increased steadily as the oogenesis progressed, reaching their maximal values in females with mature oocytes (stages 5 to 7, Fig. 1B). Conversely, no correlation was found between Vtg and Zrp1 expression levels and reproductive status in males (Table S2).

Male trout showed very low Vtg mRNA levels in essentially all cases, whereas Zrp1 levels varied by more than three orders of magnitude (Fig. 1C, Table S1). Analysis of ERa mRNA levels showed a similar variability among male specimens, as well as a strong correlation with Zrp1 mRNA levels (Fig. 1C, Table S2). These variations did not correlate with temperature or physiological parameters like age or condition factor (Table S2) and were in sharp contrast with the observed strong correlation between hepatic Cyp1A and the reciprocal of absolute temperatures, as previously reported^14^ (Table S2.) Given the vast amounts of pollutants putatively able to induce feminization, we opted to look for correlations between this effect and chemical gradients of persistent organic compounds occurring in the studied fish populations, rather than testing individual molecules. Muscle contents of hexachlorobenzene (HCB), α- and γ-hexachlorocyclohexanes (HCH), 4,4´-DDE, 4,4´-DDT and seven polychlorobiphenyl (PCB) congeners (Table S1) were analysed and their distribution among samples studied by principal component analysis (PCA). Two principal components (PC1 and PC2) explained 58% of the variability of the concentrations of these compounds, 45% for PC1 and 13% for PC2. In general terms, PC1 showed a strong contribution of PCBs, 4,4′-DDT, 4,4′-DDE and γ-HCH whereas PC2 was composed of HCB and in lower proportion some pesticides such as α- and γ-HCH, and 4,4′-DDT (Fig. 2A). No significant male-female differences in the loadings of these PCs and pollutant content were observed (Fig. 2B).

Correlation of these PC scores with the expression levels of the estrogen-responsive genes and with those of Cyp1A for comparison showed significant correlations between ER and Zrp1 and PC2 (male specimens), and between Cyp1A and PC1 (males and females, Fig. 2C; Table S2). The correlation between Cyp1A expression in liver and the contents in highly chlorinated PCB congeners both in lake sediments and fish has been reported previously^16^,^17^.

The correlation of PC2 and the expression genes of ER and Zrp1 indicates an estrogenic impact of long-range atmospheric transported chemical contamination in fish population. The strong correlation of the expression of ER and PC2 (Fig. 2C, Table S2) suggests that in this fish species the early response to feminization occurs through activation of this gene. The four to six orders of magnitude variation on the expression of estrogen-responsive genes in females during the natural reproductive cycle (Fig. 1B) precluded the analysis of any correlation between their mRNA levels and pollutant content in females.

The present results evidence a clear estrogenic impact in fish from pristine continental sites such as high mountains. They show for the first time a feminization of vertebrates that is exclusively linked to airborne pollution. In previous studies, we showed an estrogenic activity in extracts of muscle fish and sediments collected in these high mountain lakes using recombinant yeast assays^18^,^19^. These results were consistent with the present observations, e.g. positive correlations between HCB and levels of estrogenicity^18^,^19^, but they were not related to effects on the organisms living in these lakes. Now, the present mRNA results provide a quantitative assessment showing that the deposition of these atmospheric pollutants really elicit an estrogenic response in the fish from these water systems. The observed degree of feminization does not constitute a direct threat to the reproductive capacity of the impacted animals but reveals a background level of spread male feminization that reaches highly remote sites.

The present approach allows to discriminating between the effects of the studied persistent organic pollutants. The correlation of PCB, DDT and HCH contents and Cyp1A expression is related to the ectopic activation of the aryl hydrocarbon receptor by dioxin-like compounds^16^,^17^. Conversely, HCB has been described to elicit estrogenic-like responses^20^, including vitellogenin expression in male fish^21^,^22^, although its mechanisms of action seems not requiring binding to ER^23^. However, HCB may only be part of the chemical molecules responsible for the observed expression of the estrogen-regulated genes in the studied fish, e.g other organochlorine compounds also have significant loads in PC2. In any case, these compounds are usually found in the atmosphere of remote areas, including high mountains^24^ and they are widespread in the atmosphere, including the free troposphere^25^, which is consistent with the distribution of feminizing impact observed in the present study.

These organochlorine compounds are also common in humans in which birth defects^26^,^27^, obesity^28^,^29^ and thyroid metabolism alterations^30^ have been related to their potential estrogenic effects. The present results may therefore be relevant from both environmental and human health standpoints.

## Methods

### Sampling sites

The samples included in this work belonged to water bodies from two mutually distant European mountain ranges, the Tatras and the Pyrenees (Table S1). All fish were captured in mountain lakes situated in catchment receiving organic contaminants only by atmospheric transport.

### Fish sampling

Fish (*S. trutta*, brown trout) were sampled by net fishing. They were killed by cervical dislocation, weighed, measured and dissected in the sampling site, reducing any stress or undue suffering. Condition factor was calculated as K = (W/L^3^) x 100, where W = wet weight in g, and L = fork length in cm. Maturation status was determined by dissection and evaluated in a scale from 1 (undifferentiated gonads) to 7 (post-spawning). Liver samples (50–100 mg) were preserved in RNAlater (Sigma-Aldrich, St. Louis, MO, USA) immediately after dissection^14^. Muscle fillets (skinless) for chemical analysis were wrapped in pre-cleaned aluminium foil and stored at −20 °C.

### Induction experiments

Wild brown trout were captured in brooks from the Tyrolean Alps, transported to conditioned aquaria in Innsbruck University, and placed for several weeks in 100 L flow-through tanks at 19 °C under natural conditions of photoperiod. Ten brown trout juveniles (mean body weight 11.4 ± 2.6) were treated with a single dose intraperitoneal injection of estradiol (1 mg/kg) and kept for 48 h. They were sacrificed by neck dislocation and liver samples stored in RNAlater. All procedures with fish described in this study were performed in accordance with the guidelines established within the EUROLIMPACS project (GOCE-CT-2003-505540) financed by the European Union. They were also approved by the Institutional Animal Care and Use Committee of the Center for Research and Development of the Spanish Council for Scientific Research (CID-CSIC).

### mRNA analysis by RT-qPCR

Liver samples, either frozen in liquid nitrogen or preserved in RNAlater, were stored at −80 °C. Samples were homogenised in TRIzol Reagent (Gibco, Paisley, UK) using Eppendorf-fitting, RNase free pestles (Iberlabo, Madrid, Spain). RNA was extracted in TRIzol. Total RNA concentration was estimated by spectrophotometric absorption at 260 nm in a Nanodrop Spectrophotometer ND-1000 (NanoDrop Technologies; Delaware, USA) and treated with DNAse I (F. Hoffmann-La Roche Ltd, Basel, Switzerland) to remove contaminating genomic DNA. Ten μg of DNaseI-digested RNA were copied to cDNA by reverse transcriptase (Omniscript, Qiagen, Valencia, CA, USA) and stored at −20 °C. Aliquots corresponding to 40 ng of the original RNA preparation were used to quantify specific transcripts in a Abi Prism 7000 Sequence Detection System (Applied Biosystems, Foster City, CA, USA) by the SYBR GREEN method (Applied Biosystems).

The sequences were designed with PrimerExpress (Applied Biosystems), using known Salmonidae sequences. For alignment of Vitellogenin: X92804.1 (*Oncorhynchus mykiss* Vtg1 gene), AF454752.1 (*Salvelinus fontinalis* Vtg A gene), AF454751.1 (*S. alpinus* Vtg A gene), AF454748.1 (*Oncorhynchus tshawytscha* Vtg A) and AF454747.1 (*Oncorhynchus kisutch* Vtg A gene). For aligment of Zona radiata protein: AY426716.1 (*S. alpinus* zona pellucida protein beta mRNA), AF407574.1 (*O. mykiss* ZR structural protein mRNA), AF231707.1 (*O. mykiss* vitelline envelope protein beta mRNA), AJ000664.1 (*S. salar* mRNA for eggshell protein), AF231706.1 (*O. mykiss* vitelline envelope protein alpha mRNA) and AY426715.1 (*S. alpinus* zona pellucida protein alpha mRNA). For aligment of the Estrogen Receptor: DQ248228.1 (*O. kisutch* ERα 1); DQ009007.2 (*S. salar* ERα), AJ242741.1 (*O. mykiss* mRNA for ER, short isoform), AJ242740.1 (*O. mykiss* mRNA for ER, long isoform), AY520443.2 (*O. masou* estrogen ERα), DQ177438.1 (*O. mykiss* ERα 2), DQ177439.1 (*O. mykiss* ERβ 1 mRNA) and AY508959.1 (*S. salar* ERβ mRNA). The final primer sequences are shown in Supplementary Table S3.

The relative amounts of cDNA in the samples were calculated from the number of cycles required for amplification reaction to reach fluorescence above the threshold level in the RT-qPCR reaction (CT values), according to equation 1:
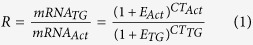
where EAct and ETG correspond to Real-Time efficiency for ß-actin^16^ and the target gene, respectively. Efficiency values for all tested primers were initially found to be close to 100%. Thus, EAct and ETG were assigned as 1 for all further calculations. The results were expressed as copies of a given gene mRNA per 1000 copies of ß-actin mRNA. Typical experiments calculated average CT values from three replicates.

### Chemical analysis

The muscle samples were ground with activated sodium sulphate. The mixtures were Soxhlet extracted with (4:1) n-hexane:dichloromethane (18 h) in cellulose cartridges after addition of TBB and PCB209 standards. The extracts were cleaned up with sulfuric acid (5 times), concentrated by vacuum rotatory evaporation and to near dryness under a gentle nitrogen flow. Finally, they were redissolved in isooctane (50 μl). PCB142 was added as internal standard before instrumental analysis. Samples were analyzed by GC-ECD and GC-MS with a 60 m × 0.25 mm i.d. DB-5 capillary column (J&W Scientific, Folsom, CA) coated with 5% phenyl/95% methylpolysiloxane (film thickness 0.25 μm). The GC operated in splitless mode. The oven temperature program started at 90 °C (held for 2 min), ramped to 150 °C at 15 °C min^−1^ and to 290 °C at 4 °C min^−1^ (holding time 20 min). Injector and detector temperatures were 280 and 310 °C, respectively. Helium and nitrogen were used as carrier (1.5 mL min^−1^) and makeup (60 mL min^−1^) gases, respectively.

### Statistics

The SPSS v. 19.0.0 package (SPSS Inc., Chicago, Ill.) was used in all statistical calculations. Physical, chemical and biological data were compared using the non-parametric Spearman test. Principal Component Analyses were performed after logarithmic transformations of mRNA abundances and chemical concentrations.

## Additional Information

**How to cite this article**: Jarque, S. *et al.* Background fish feminization effects in European remote sites. *Sci. Rep.* **5**, 11292; doi: 10.1038/srep11292 (2015).

## Supplementary Material

Supplementary Information

## Figures and Tables

**Figure 1:**
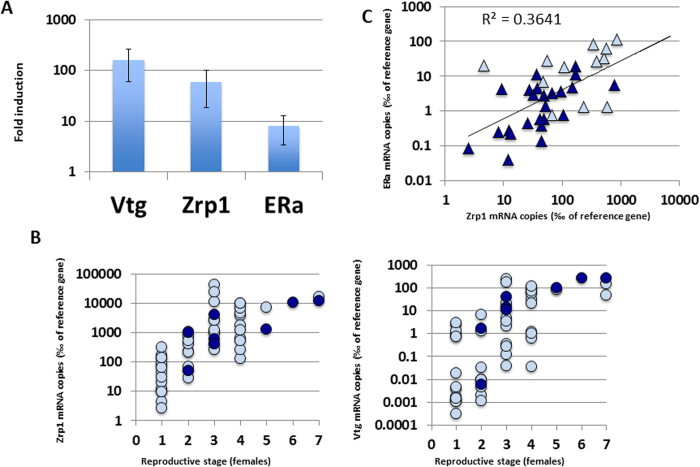
Analysis of hepatic mRNA levels of estrogen-responsive genes in European high mountain lakes. **A**) Fold induction in mRNA levels for Vtg, Zrp1 and ERα in estradiol-treated juvenile trouts. Data represents average fold induction and standard deviations. **B**) Correlation of Vgt (left) and Zrp1 (right) mRNA levels in *S. trutta* females from Pyrenees (pale blue) and Tatras (dark blue) with reproductive stage (1- undifferentiated, 7- mature oocytes/spawning). **C**) Double log correlation between ERα and Zrp1 mRNA levels in males from Pyrenees (pale blue) and Tatras (dark blue). The corresponding linear regression and the correlation coefficient are shown.

**Figure 2:**
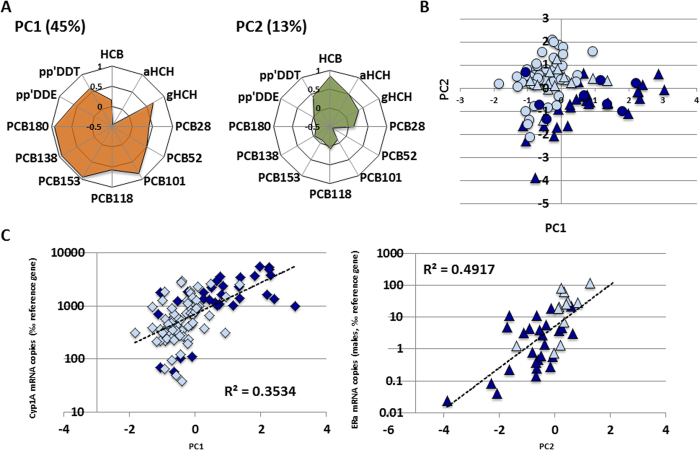
Results of Principal Component Analysis. **A**) Loading plots for PC1 and PC2; the explained variation for each PC is indicated. **B**) Score plot for PC1 and PC2. Males and females are indicated by triangles and circles, respectively. Specimens from Pyrenees and Tatras are indicated by pale and dark blue symbols, respectively. **C**) Correlations between PC1 (left) and PC2 (right) scores and Cyp1A (all fish) and ERα mRNA levels (males), respectively. The corresponding linear regression (semi-log transformation) and the correlation coefficients are shown.
