# Supplementary material for: Background fish feminization effects in European remote sites
Source: Sci Rep. 2015 Jun 10;5:11292. doi: 10.1038/srep11292 (PMC4462152; doi:10.1038/srep11292)
Supplement: Supplementary Information [file srep11292-s1.pdf]

Supplementary information

Background fish feminization effects in European remote sites

Sergio Jarque[1], Laia Quirós[1], Joan O. Grimalt[1*], Eva Gallego[1], Jordi Catalan[2], Reinhard Lackner[3], Benjamin Piña[1].

[1]Institute of Environmental Assessment and Water Research (IDÆA-CSIC). Jordi Girona, 18. 08034 Barcelona, Catalonia, Spain, [2]CREAF. Edifici C, Campus de Bellaterra. Autonomous University of Barcelona. 08193 Cerdanyola del Vallès, Catalonia, Spain, [3]Institute of Zoology, University of Innsbruck, Technikerstrasse 25, Innsbruck, A-6020, Austria. *e-mail: joan.grimalt@idaea.csic.es

Table S1. Averaged data of the different parameters analyzed in this study, grouped by fish population

Zone	Lake	Altitude	Calculated T		*n*	Lenght	Weight	CF	RS	Age	Vtg mRNA levels	Zrp1mRNA levels	ER mRNA levels	Cyp1A mRNA levels	HCB	g-HCH	Sum PCB	SUM DDE
		(m)	(ºC)		*n*	(cm)	(g)	(cg cm^{-3})	(a)	(yr)	(b)	(b)	(b)	(b)	(pg/g)	(pg/g)	(pg/g)	(pg/g)
Pyrenees	Llebreta	1620	6.11	All	14	32 ± 3	347 ± 100	1.1 ± 0.1	2.6 ± 1.8	6.2 ± 1.4	20 ± 38	2400 ± 4600	2.2 ± 1.0	270 ± 120	0.36 ± 0.15	1.0 ± 1.1	4.2 ± 1.0	5.1 ± 1.5
	42.5508N 0.8903E			Males	1	36	560	1.2	1.0	6.0	b.d.l.	590	1.1 .	440	0.13	0.7	5.9	6.9
				Females	13	31 ± 3	330 ± 83	1.1 ± 0.1	2.8 ± 1.8	6.2 ± 1.5	21 ± 39	2600 ± 4800	2.3 ± 0.9	260 ± 110	0.37 ± 0.14	1.1 ± 1.2	4.0 ± 0.9	5.0 ± 1.5
	Cavallers	1800	5.50	All	14	27 ± 4	266 ± 104	1.2 ± 0.1	2.7 ± 1.4	4.5 ± 1.2	15 ± 25	1200 ± 1500	1.9 ± 0.9	550 ± 700	0.49 ± 0.13	1.0 ± 0.5	4.9 ± 1.1	4.3 ± 1.1
	42.5926N 0.8578E			Males	4	30 ± 2	346 ± 74	1.3 ± 0.0	1.0 ± 0.0	4.5 ± 1.0	0.8 ± 1.2	87 ± 100	1.5 ± 0.9	1000 ± 1300	0.46 ± 0.11	1.1 ± 0.3	5.1 ± 1.0	4.0 ± 0.9
				Females	10	26 ± 5	235 ± 99	1.2 ± 0.1	3.4 ± 1.1	4.5 ± 1.4	19 ± 28	1600 ± 1600	2.5 ± 0.9	370 ± 200	0.50 ± 0.14	1.0 ± 0.6	4.8 ± 1.2	4.5 ± 1.2
	Llong	2000	4.10	All	10	27 ± 4	254 ± 116	1.2 ± 0.1	1.7 ± 1.3	8.0 ± 2.4	19 ± 42	2100 ± 4200	2.9 ± 0.7	490 ± 360	0.29 ± 0.10	1.3 ± 0.6	5.7 ± 4.3	4.9 ± 2.2
	42.5743N 0.9506E			Males	2	24 ± 1	165 ± 35	1.2 ± 0.0	1.0 ± 0.0	7.0 ± 2.8	b.d.l.	77 ± 42	2.1 ± 0.4	150 ± 120	0.40 ± 0.11	1.7 ± 0.2	4.9 ± 2.3	5.4 ± 2.8
				Females	8	28 ± 4	276 ± 120	1.2 ± 0.1	1.9 ± 1.4	8.3 ± 2.4	24 ± 47	2600 ± 4600	3.2 ± 0.6	580 ± 350	0.26 ± 0.08	1.2 ± 0.6	5.9 ± 4.8	4.8 ± 2.2
	Redo	2235	3.18	All	7	27 ± 5	204 ± 81	1.0 ± 0.1	n.d.	9.9 ± 3.8	7.6 ± 8.1	2100 ± 2500	3.0 ± 0.4	1700 ± 700	0.52 ± 0.11	2.6 ± 2.1	18 ± 13	16 ± 14
	42.6421N 0.7795E			Males	5	26 ± 7	195 ± 95	0.9 ± 0.1	n.d.	9.0 ± 4.2	3.8 ± 5.1	1900 ± 2900	3.0 ± 0.4	1900 ± 500	0.56 ± 0.10	3.4 ± 1.6	22 ± 13	19 ± 15
				Females	2	27 ± 2	228 ± 41	1.1 ± 0.1	n.d.	12 ± 1.4	17 ± 7	2800 ± 1600	n.d.	1100 ± 1000	0.39 ± 0.00	0.2 ± 0.0	6.3 ± 0.0	7.6 ± 0.0
	Xic de Colomina	2425	2.50	All	10	24 ± 2	148 ± 36	1.1 ± 0.2	1.7 ± 0.9	6.2 ± 0.8	3.0 ± 4.1	5500 ± 13900	1.5 ± 0.7	1400 ± 800	0.40 ± 0.14	1.6 ± 0.8	8.2 ± 4.5	9.9 ± 5.0
	42.5215N 0.9956E			Males	0	n.d.	n.d.	n.d.	n.d.	n.d.	n.d.	n.d.	n.d.	n.d.	n.d.	n.d.	n.d.	n.d.
				Females	10	24 ± 2	148 ± 36	1.1 ± 0.2	1.7 ± 0.9	6.2 ± 0.8	3.0 ± 4.1	5500 ± 13900	1.5 ± 0.7	1400 ± 800	0.40 ± 0.14	1.6 ± 0.8	8.2 ± 4.5	9.9 ± 5.0
	Vidal d'Amunt	2688	1.00	All	9	29 ± 3	279 ± 55	1.1 ± 0.2	2.8 ± 1.4	5.4 ± 1.2	52 ± 91	3000 ± 7100	3.8 ± 0.9	840 ± 350	0.65 ± 0.30	2.2 ± 0.5	9.8 ± 8.0	19 ± 8
	42.5328N 0.9935E			Males	3	29 ± 2	266 ± 28	1.1 ± 0.2	2.0 ± 0.0	5.0 ± 0.0	0.02 ± 0.01	400 ± 300	3.6 ± 0.7	640 ± 270	0.54 ± 0.12	2.1 ± 0.3	6.5 ± 1.7	16 ± 4
				Females	6	29 ± 3	282 ± 62	1.1 ± 0.2	3.1 ± 1.5	5.6 ± 1.3	69 ± 100	4200 ± 8500	4.1 ± 1.2	930 ± 330	0.70 ± 0.33	2.2 ± 0.5	11 ± 9	20 ± 8
Tatras	Morskie Oko	1395	2.40	All	13	21 ± 10	245 ± 649	1.1 ± 0.7	2.9 ± 2.4	7.1 ± 3.7	48 ± 106	2100 ± 4600	1.1 ± 0.7	910 ± 890	0.18 ± 0.18	1.2 ± 0.7	55 ± 170	110 ± 360
	49.1978N 20.0722E			Males	10	22 ± 12	295 ± 741	1.0 ± 0.3	2.3 ± 2.0	7.5 ± 4.1	0.10 ± 0.23	73 ± 82	1.1 ± 0.7	1200 ± 900	0.17 ± 0.19	1.1 ± 0.7	70 ± 193	140 ± 410
				Females	3	18 ± 4	75 ± 8	1.7 ± 1.3	5.0 ± 2.6	5.7 ± 0.6	175 ± 152	7600 ± 6600	n.d.	77 ± 24	0.22 ± 0.17	1.4 ± 0.5	2.8 ± 1.8	14 ± 9
	Popradske Pleso	1494	2.00	All	13	19 ± 3	73 ± 42	0.9 ± 0.1	3.4 ± 2.1	7.2 ± 2.4	5.1 ± 11.7	450 ± 1100	1.5 ± 0.7	2700 ± 1800	0.40 ± 0.17	4.0 ± 1.7	240 ± 130	16 ± 13
	49.1536N 20.0799E			Males	10	19 ± 3	79 ± 47	1.0 ± 0.1	3.4 ± 2.4	7.1 ± 2.7	0.02 ± 0.06	58 ± 50	1.5 ± 0.7	2900 ± 1900	0.43 ± 0.18	3.8 ± 1.1	250 ± 150	18 ± 14
				Females	3	19 ± 1	53 ± 3	0.8 ± 0.1	3.3 ± 0.2	7.3 ± 1.2	22 ± 16	1600 ± 2000	n.d.	2200 ± 1200	0.30 ± 0.12	4.9 ± 3.2	190 ± 50	9.3 ± 3.4
	Velke Hinovo	1946	-0.70	All	10	16 ± 1	46 ± 12	1.0 ± 0.1	3.1 ± 1.4	5.9 ± 1.0	10 ± 32	340 ± 490	1.0 ± 0.4	1700 ± 800	0.20 ± 0.09	1.5 ± 0.5	17 ± 7	25 ± 15
	49.1797N 20.0606E			Males	8	16 ± 1	44 ± 11	1.1 ± 0.1	3.0 ± 1.3	5.5 ± 0.5	0.04 ± 0.04	140 ± 260	1.0 ± 0.4	1900 ± 800	0.18 ± 0.07	1.4 ± 0.5	16 ± 5	23 ± 13
				Females	2	18 ± 1	56 ± 15	1.0 ± 0.0	3.5 ± 2.1	7.5 ± 0.7	52 ± 72	1100 ± 200	n.d.	1000 ± 5	0.30 ± 0.11	2.1 ± 0.2	22 ± 11	30 ± 27

CF. Conditioning factor

RS. Reproductive status

b.d.l., below detection limits; n.d., no data available

a) 1: undifferentiated gonads, 7: post-spawning

b) mRNA copies per 100 copies of b-Actin mRNA

Table S2. Spearman rank correlations (ρ) between hepatic mRNA levels for different genes and physical and biological parameters and pollutant content.

	Cyp1A		Vtg				Zrp1				ERa			
	Males and females		Males		Females		Males		Females		Males		Females	
	ρ	*n*	ρ	*n*	ρ	*n*	ρ	*n*	ρ	*n*	ρ	*n*	ρ	*n*
Cyp1A	1	101												
Vtg	0.334 *	39	1	39	1	51								
Zrp1	-0.013	39	0.172	37	0.659 * * *	49	1	39	1	50				
ERa	-0.208	38	0.050	37	-0.395	24	0.584 * * *	36	0.779 * * *	25	1	38	1	25
Temperature	-0.555 * * *	101	0.041	39	-0.171	51	0.150	39	0.026	50	0.125	38	-0.020	25
Condition factor	-0.283 * *	101	0.126	39	-0.089	51	-0.007	39	-0.149	50	-0.028	38	0.314	25
Reproductive status	-0.094	94	-0.102	34	0.669 * * *	49	-0.364 *	34	0.787 * * *	48	-0.107	35	0.243	25
Age	0.017	100	-0.175	38	0.121	51	0.278	38	0.128	50	0.005	37	-0.187	25
a-HCH	-0.323 * *	96	0.333	35	0.153	50	0.244	35	0.180	49	0.226	36	0.152	25
HCB	-0.108	98	-0.013	37	0.285 *	50	0.211	37	0.376 * *	49	0.590 * * *	38	0.267	25
g-HCH	0.385 * * *	98	-0.175	37	0.078	50	0.066	37	-0.041	49	0.274	38	-0.054	25
PCB28	0.385 * * *	98	0.250	37	0.272	50	-0.048	37	0.145	49	0.002	38	-0.002	25
PCB52	0.295 * *	94	-0.049	34	0.168	50	0.123	33	0.218	49	0.252	35	-0.044	25
PCB101	0.506 * * *	95	-0.013	35	0.111	50	-0.017	34	0.162	49	-0.013	35	0.106	25
PCB118	0.432 * * *	98	0.034	37	0.150	50	0.267	37	-0.150	49	0.143	38	-0.328	25
PCB153	0.575 * * *	98	-0.191	37	-0.024	50	0.019	37	-0.245	49	0.005	38	-0.219	25
PCB138	0.579 * * *	98	-0.130	37	-0.044	50	0.071	37	-0.303 *	49	0.120	38	-0.161	25
PCB180	0.578 * * *	98	-0.153	37	-0.021	50	-0.095	37	-0.265	49	-0.157	38	-0.387	25
Sum PCB	0.633 * * *	98	-0.156	37	0.022	50	0.021	37	-0.259	49	0.009	38	-0.217	25
pp-DDE	0.437 * * *	98	0.141	37	0.054	50	0.150	37	-0.177	49	0.043	38	-0.210	25
pp_DDT	0.428 * * *	95	0.053	34	0.128	50	0.210	34	-0.052	49	0.220	35	-0.322	25
SUM DDE	0.459 * * *	98	0.137	37	0.050	50	0.141	37	-0.191	49	0.057	38	-0.215	25
PC1	0.583 * * *	98	-0.089	37	0.170	50	0.032	37	-0.129	49	0.084	38	-0.213	25
PC2	-0.194	98	0.108	37	0.228	50	0.341 *	37	0.212	49	0.669 * * *	38	0.071	25

*, p<0.05; **,p<0.01; ***, p<0.001

PC1, PC2, principal components 1 and 2
